# Dabigatran and Wet AMD, Results From Retinal Pigment Epithelial Cell Monolayers, the Mouse Model of Choroidal Neovascularization, and Patients From the Medicare Data Base

**DOI:** 10.3389/fimmu.2022.896274

**Published:** 2022-06-17

**Authors:** Tanjina Akter, Balasubramaniam Annamalai, Elisabeth Obert, Kit N. Simpson, Bärbel Rohrer

**Affiliations:** ^1^ Department of Ophthalmology, Medical University of South Carolina, Charleston, SC, United States; ^2^ Department of Healthcare Leadership and Management, Medical University of South Carolina, Charleston, SC, United States; ^3^ Department of Neurosciences, Medical University of South Carolina, Charleston, SC, United States; ^4^ Ralph H. Johnson VA Medical Center, Division of Research, Charleston, SC, United States

**Keywords:** VEGF, CTGF, complement, retinal pigment epithelium, mouse CNV, MarketScan, dabigatran, thrombin

## Abstract

**Background:**

Age-related macular degeneration (AMD), the leading cause of irreversible blindness in elderly Caucasian populations, includes destruction of the blood-retina barrier (BRB) generated by the retinal pigment epithelium-Bruch’s membrane complex (RPE/BrM), and complement activation. Thrombin is likely to get access to those structures upon BRB integrity loss. Here we investigate the potential role of thrombin in AMD by analyzing effects of the thrombin inhibitor dabigatran.

**Material and Methods:**

MarketScan data for patients aged ≥65 years on Medicare was used to identify association between AMD and dabigatran use. ARPE-19 cells grown as mature monolayers were analyzed for thrombin effects on barrier function (transepithelial resistance; TER) and downstream signaling (complement activation, expression of connective tissue growth factor (CTGF), and secretion of vascular endothelial growth factor (VEGF)). Laser-induced choroidal neovascularization (CNV) in mouse is used to test the identified downstream signaling.

**Results:**

Risk of new wet AMD diagnosis was reduced in dabigatran users. In RPE monolayers, thrombin reduced TER, generated unique complement C3 and C5 cleavage products, led to C3d/MAC deposition on cell surfaces, and increased CTGF expression *via* PAR1-receptor activation and VEGF secretion. CNV lesion repair was accelerated by dabigatran, and molecular readouts suggest that downstream effects of thrombin include CTGF and VEGF, but not the complement system.

**Conclusions:**

This study provides evidence of association between dabigatran use and reduced exudative AMD diagnosis. Based on the cell- and animal-based studies, we suggest that thrombin modulates wound healing and CTGF and VEGF expression, making dabigatran a potential novel treatment option in AMD.

## Introduction

Age-related macular degeneration (AMD) is associated with an irreversible destruction of the macula and is the leading cause of visual impairment and irreparable blindness among the older population in the Western world (1–3). Around 196 million people worldwide and 11 million in the United States are experiencing either form of AMD, dry (non-exudative) or wet (exudative). The overall prevalence of the disease is expected to be nearly double by 2050, therefore, imposing a substantial burden on the health care system (4, 5). The two forms of AMD share characteristics, such as the destruction of the blood-retinal barrier (BRB), development of drusen, activation of immune reposes, and deposition of complement components in the retinal pigment epithelium-Bruch’s membrane complex (RPE/BrM) (6, 7). In the dry form of the disease, these damages can progress to geographic atrophy (GA) that leads to destruction of RPE and choriocapillaris followed by loss of the overlying photoreceptors, whereas in the wet form they ultimately lead to the formation of VEGF-dependent choroidal neovascularization (CNV). In both cases, with the destruction of the BRB, blood-derived factors are likely to get access to the retina and the apical side of the RPE.

One of the blood-derived systems that has gained a lot of attention in AMD is the complement system. The complement system is a major component of the innate and adaptive immune system. Our laboratory and others have provided evidence in histological and functional studies of the deleterious effects of complement activation and deposition in on RPE health, as well as AMD risk and progression (8–12). Additionally, genome-wide association study on European-American descent complied significant evidence between factor H polymorphism and increase disease risk of AMD (13). The complement system or cascade is characterized by the generation of a sequential set of proteases that generate biological effector molecules. In short, it is triggered by three distinct pathways: the classical pathway (CP), lectin pathway (LP), and alternative pathway (AP), which all contribute to the formation of C3 convertases that cleave the complement component 3 (C3) into C3a and C3b. C3b then participates in the formation of a C5 convertase that cleaves complement component 5 (C5) into C5a and C5b. Finally, C5b initiates the formation of the membrane attack complex (MAC) on membranes resulting in sublytic cell signaling or cell lysis (3). C3a and C5a are anaphylatoxins that participate in different mechanisms, including enhancing vascular permeability and mediating chemotaxis and inflammation (4), by binding to their receptors C3aR, C5aR and C5L2 (5).

Another blood-derived system is the coagulation system, with its key enzyme thrombin (coagulation factor II). Thrombin’s main role is to convert soluble fibrinogen into insoluble strands of fibrin as part of the clotting cascade. However, in different models, it is also a known regulator of the destruction of the BRB, it promotes vascular endothelial growth factor (VEGF) secretion, and plays a role in ER-stress induction (14–16). Important in the context of our study, in C3-deficient mice, thrombin can substitute for the C3-dependent C5 convertase, generating C5a fragments, that are effective as anaphylatoxins (17). Also, thrombin has been shown to uniquely cleave the complement component C5 *in vitro*, supporting the terminal complement cascade, and resulting in erythrolytic activity (18). Proteomics analysis by LC-MS/MS showed that vitreous fluid obtained from AMD patients contains higher amounts of prothrombin compared with healthy controls (19). While no mutation has yet been reported as a risk factor for AMD, two single nucleotide polymorphisms (SNPs), the A-allele of factor V Leiden 1691 or the prothrombin 20210 gene have been found to expose the wet AMD carriers to a higher risk of failing therapeutic effectiveness of the photodynamic therapy with verteporfin (20).

AMD is a major cause of blindness in the Western world, and one of the reasons is the continued lack of therapeutics other than anti-VEGF medications to block or cure the disease. In addition, a lack of understanding of the pathogenesis of the disease limits novel drug target identification and treatment options offered to the patients. We posit that in order to expand the effective treatment options, understanding of the interrelationship or cross-talk between different signaling pathways is of paramount importance. The roles of complement and VEGF in AMD pathogenesis and progression are well studied and accepted as therapeutic targets. Some of the characteristics of AMD, such as, BRB destruction and VEGF secretion as well as production of anaphylatoxins fit well within the known physiological functions of thrombin. However, little is known about the pathological effects of thrombin in complement activation, VEGF-secretion, and physiological consequences.

This current study was undertaken to investigate the potential role of thrombin in age-related macular degeneration, by examining the use of dabigatran (thrombin inhibitor) in multiple paradigms. Dabigatran, a direct thrombin-inhibitor (New Drug Application: 022512) and anticoagulant is used for the prevention and treatment of thrombosis (21). Specifically, we first asked whether a correlation exists between dabigatran use and AMD through use of retrospective data from the MarketScan^®^ medical billing record database for Medicare patients. These results were followed up in the mouse model of wet AMD, in which laser photocoagulation of BrM is used to trigger angiogenesis and fibrosis (22, 23); and potential mechanisms of action were further explored in ARPE-19 cell monolayers. Overall, our results suggest that subjects who received dabigatran had longer time elapsing than the control population before they had the first recorded diagnosis of AMD. In the mouse model of wet AMD, dabigatran reduced CNV lesion size and accelerated repair processes, a process that seems to involve connective tissue growth factor (CTGF), a known regulator of fibrosis and signaling of growth factors, including VEGF. The potential cross-talk with the complement system is investigated. Overall, our results suggest that therapeutic intervention with the direct thrombin inhibitor-dabigatran would ameliorate disease outcomes in wet AMD.

## Material and Methods

### MarketScan Analysis

#### Population

A total of 41,860 dabigatran-exposed and 41,860 non-exposed controls, for a total of 83,720 Medicare patients were extracted from MarketScan^®^ data from 2010-2015 and used in the analysis. Patients with an inpatient or outpatient record with a diagnosis of AMD (wet or dry) for the baseline period were excluded from inclusion in the data set. Patients who filled at least one prescription of dabigatran during the 2011-2012 exposure period were included in the dabigatran group. The date of the first filled prescription was designated as the Index Date. A potential control group of 1,000,000 patients who had no records of dabigatran prescriptions during the baseline or exposure period were randomly selected from outpatient utilization records. The first patient record date in randomly selected records from 2011 or 2012 was chosen as the Index Date for control group patients. Patients with <90 days of insurance coverage in the baseline period or <120 days of coverage in the study (exposure) period were excluded from the final cohort. The exposed and control patients were matched by age, sex, state of residence, Index Date, days in baseline period, and days in study period using 1:1 propensity score mating with a greedy algorithm and a caliper distance of 0.2. A total of 41,860 exposed patients and similar group of controls selected from a pool of 122,636 patients were matched within the matching specification.

#### Survival and Statistical Analysis

Survival analysis was used according to our previous study (24) to show differences in time until AMD diagnosis among dabigatran users and control subjects using the LIFETEST procedure. AMD diagnoses were identified using ICD-9 codes 362.5 (unspecified macular degeneration), 362.51 (dry AMD), and 362.52 (wet AMD). A period of ~5 years (from July 1, 2010, to December 31, 2015) was used for this analysis, and preexisting AMD diagnosis was ruled out by requiring that the subjects be AMD free over the first ~600 days (designated as baseline days or Base Days). Data on the matched control and dabigatran population were analyzed using multivariable logistic and Cox regression. Specifically, we compared the odds of being diagnosed with AMD during the follow-up time, controlling for days of dabigatran exposure, and the proportional hazard rates of obtaining an AMD diagnosis in the dabigatran or control population as well as any baseline differences in demographics or comorbidities (Charlson or Elixhouser diagnoses).

### Mouse Model of Choroidal Neovascularization (CNV)

#### Choroidal Neovascularization

C57BL/6J mice (males and females) from our own colony, to guarantee identical microbiome (25), were recruited to the study at 3 months of age. Argon laser photocoagulation (532 nm, 100 µm spot size, 0.1 s duration, 100 mW) was used to generate 4 laser spots around the optic nerve of each eye (22), utilizing bubble formation at the site of the laser burn as the inclusion criteria for successful Bruch’s membrane rupture (26).

#### Dabigatran Exposure

Mice were fed standard chow fortified with dabigatran etexilate (10 μg/g of chow) which was provided by Boehringer Ingelheim. Mice were either fed dabigatran chow during the angiogenesis period of CNV (starting 2 days prior to CNV induction through day 6 when the tissues were collected), or during the wound healing period (starting after the OCT analysis to ensure CNV growth on day 5 through day 23).

#### Optical Coherence Tomography (OCT) Analysis

OCT was used to quantify CNV lesion size on day 5 post laser treatment (27–29), or in weekly intervals to document recovery from fibrosis (23) using an SD-OCT Bioptigen^®^ Spectral Domain Ophthalmic Imaging System (Bioptigen Inc., Durham NC). Mice were anesthetized prior to imaging, eyes kept hydrated with normal saline, and body temperature was carefully monitored to prevent anesthesia induced cataracts. Our methods for imaging and analysis have been carefully described in other publications (23, 27–29). Based on the size of the individual pixels (1.6 x 1.6 µm), the lesion sizes were calculated.

### Cell Culture Experiments

#### Cells

Human ARPE-19 (ATCC^®^ CRL-2302™; American Type Culture Collection, Manassas VA) cells were cultured in DMEM cell culture media (Gibco/ThermoFisher Scientific) containing high glucose DMEM with D-glucose (4.5 g/L), L-glutamine, sodium pyruvate (110 mg/L), Penicillin and Streptomycin (1X) and 10% of FBS. Cells (< passage 10 after purchase) were expanded in T75 cell culture flasks at 37°C, in the presence of 5% CO_2_. Confluent cells were trypsinized with 0.05% trypsin (Gibco), and equal numbers of cells were seeded on 6-well transwell filters/plate (Costar). After confluent monolayers of cells were developed the percentage of FBS was gradually decreased from 10%, 2%, to 1% to promote cell differentiation and tight junction formation (30). Integrity of the cell monolayer was assessed by Transepithelial resistance (TER) measurements using an EVOM volt-ohmmeter (World Precision Instruments) four weeks after plating. RPE monolayers with a stable TER repeatedly measured as ~40–45 Ωcm^2^ were used for these experiments. In addition to TER, we have previously confirmed the integrity of the monolayers by demonstrating the presence of β-actin filament distribution in the form of circumferential bundles, the presence of two cell-junction markers at the cell-borders, ZO-1 and occludin, and co-labeling of ZO-1 and phalloidin (31), and Balmer and colleagues report the presence of RPE65 in four-week-old ARPE-19 cell monolayers on transwell plates (32).

#### Treatments

Prior to each experiment, the cell monolayer was washed with serum-free medium (SFM) and maintained in SFM for 24 hours. During that time, RPE cells secrete proteins, including complement components into the supernatant (33–35). All treatments were performed on the apical side of the transwell membrane, which represents the retinal side of the RPE *in vivo*. Some of the cells were treated with thrombin (EMD Millipore Corp), complement components C3a, C5a (Complement Technology), PAR1 agonist PAR1-AP (Sigma-Aldrich; amino acid sequence SFLLRN), thrombin inhibitor dabigatran etexilate (Sigma-Aldrich), C3 inhibitor compstatin (R&D Systems), AP inhibitor TT30 (generously provided by Alexion Therapeutics), a protease inhibitor alpha1-antitrypsin (Sigma-Aldrich), and the thrombin receptor proteinase activated receptor 1 inhibitor SCH79797 (Sigma-Aldrich). Treatments were performed 60 min prior to the addition of thrombin, and concentrations are indicated in the text. Cell culture supernatant and lysate were collected after specific time points or after 24 hrs of treatment and stored at -20°C for later use.

### Western Analysis

Mouse RPE/choroid/sclera (from herein referred to as RPE/choroid fraction) preparations were extracted as described previously (36). Apical supernatants from ARPE-19 cells were concentrated using Amicon Ultra-4 centrifugal filters (EMD Millipore) at 4°C. Cell lysates were collected in RIPA cell lysis buffer containing 150 mM NaCl, 1.0% IGEPAL^®^ CA-630, 0.5% sodium deoxycholate, 0.1% SDS, 50 mM Tris (pH 8.0) (Sigma-Aldrich) in presence of 1X protease inhibitor (Sigma-Aldrich). Equal amount of each samples were loaded on 4-20% Criterion™ TGX™ precast gels (Bio-Rad Laboratories, Inc.) as described previously (37). Proteins were transferred to a polyvinylidene difluoride and incubated in primary antibody followed by appropriate secondary antibodies coupled to peroxidase, followed by band development and detection using Clarity™ Western ECL blotting substrate (Bio-Rad Laboratories, Inc.) and chemiluminescent detection. Protein bands were scanned and densities quantified using ImageJ software. The following antibodies were used: C3a (1:1000; Complement Technology), C5a (1:1000; Abcam), connective tissue growth factor/CTGF (1:1000; Abcam), C3d anti-C3d (clone 11, 1:1000; generation of our antibody is described in (38)), VEGF (1:1000, Santa Cruz Biotechnology, Inc.), β-actin (1:2000; Cell Signaling Technology) and concentrations selected based on the data sheet recommendations.

### Thrombin Activity Assay

Thrombin activity was measured according to a methodology described by Ludwicka with modifications (39). Thrombin specific peptide Boc-Val-Pro-Arg-7-amido-4-methylcoumarin hydrochloride (Sigma-Aldrich) was reconstituted in molecular grade ice-cold H_2_O at a concentration of 0.5 mg/ml and used as substrate. To measure the level of active thrombin in serum or tissue extracts, an aliquot (100 µl) was mixed with 50 µl of assay buffer (50 mM Tris, 100 mM NaCl, and 0.01% BSA; pH 7.5) and 50 µl of thrombin substrate at 37°C. Absorbance was read on a spectrophotometer at 405 nm using a Biotek Synergy HT Microplate Reader and thrombin activity was determined by extrapolation from a thrombin standard curve.

### Immunofluorescence Staining

ARPE-19 cells were grown on 6 well transwell plates as described (33), treated with thrombin and inhibitors, and fixed in 4% paraformaldehyde for 15 min at room temperature. Primary antibodies against C5b-9 (1:200, Abcam) and C3d (1:100) (38) were diluted in blocking buffer (10% normal goat serum, 3% BSA, 0.4% Triton X-100 in PBS) and cells were incubated for overnight at 4°C, followed by washes and incubation with goat anti-rabbit Alexa Fluor^®^ 568 –conjugated IgG or donkey anti-goat Alexa Fluor^®^ 488-conjugated secondary antibodies (1:500; Thermo Fisher Scientific), respectively. Nuclei were identified with DAPI (Sigma-Aldrich). Transwell membranes were covered with aqua-mount mounting media (Thermo Scientific), cover slipped and photographed (Olympus 1X73 Research Inverted Microscope equipped with cellSens imaging software).

### VEGF ELISA

VEGF in the supernatant was measured using a Quantikine^®^ Human VEGF Immunoassay (R & D System) according to the manufacturer’s protocol. In brief, 200 μl of standard/sample was mixed with 50 μl of assay diluent and added to the plate precoated with the capture antibody. After washing, 200 μl of the VEGF detection antibody (polyclonal antibody specific for human VEGF conjugated to horseradish peroxidase) was added, followed by freshly prepared substrate reagents (stabilized hydrogen peroxide and tetramethylbenzidine). The reaction was terminated using 50 μl of stop solution and absorbance was measured at λ1 450 nm with wavelength correction λ2 at 540 nm. VEGF concentration of samples were calculated comparing the absorbance with the standards and converted into fold change considering the serum-free condition as 1 in the scale.

### Statistics

For data consisting of multiple groups, one-way ANOVA followed by Fisher’s *post hoc* test (*P <*0.05) was used; single comparisons were analyzed by *t* test analysis (*P <*0.05).

## Results

### Association Between Dabigatran Use and AMD Risk

The patient population consisted of 83,720 Medicare patients extracted from MarketScan^®^ data from 2010-2015 as described in Material and Methods, with 41,860 dabigatran-exposed and 41,860 non-exposed controls. The controls were matched on 33 baseline variables, including demographics and clinical characteristics (24) (**Table 1**). Patients who received dabigatran had a longer time elapsing than the control population (P< 0.0001) before they had the first recorded diagnosis of unspecified AMD (hazard ratio 0.63). Overall, the percent subjects with an AMD diagnosis was 13.95% in the control group, and reduced to 9.1% in the dabigatran group, with the percent AMD per study year being reduced by ~35%. Specifically, the percent patients with a wet AMD diagnosis was reduced by ~60% (control 2.29; dabigatran 0.91) which is reflected in the percent wet AMD per study year (control 0.68; dabigatran 0.28) (**Table 2**). The percent wet AMD per study year was not affected by the number of years of dabigatran exposure (< 1 year 0.28; 1-2 years 0.26; > 2 years 0.29). Survival analysis of days to first AMD event show similar findings. Multivariable logistic regression models controlling for age, sex and days in the study showed an odds ratio (OR) of 0.58 (confidence interval CI 0.55-0.61) of AMD for the dabigatran group compared to controls, with risk of AMD by exposure strata of 0.52 for <1 year, 0.64 for 1-2 years and 0.64 for 2+ years of dabigatran compared to controls.

**Table 1:** Characterization of control and dabigatran-exposed subjects.

Dabi	n obs	Variable	n	Mean	std dev
no	41860	age nAMD days aAMD days days to AMD dabi days base days study days AMD days	41860 36865 41015 5840 41860 41860 41860 41860	75.3 1041 982 387 0 606 1089 967	7.5 624.7 629.8 380.7 0 300.2 616.1 634.3
yes	41860	age nAMD days aAMD days days to AMD dabi days base days study days AMD days	41860 38399 41511 3810 41860 41860 41860 41860	76.2 1047 1011 562 398 612 1080 1007	6.9 619.5 619.6 417.3 327.0 270.7 620.4 619.7

41860 subjects were identified per group and characterized for age, baseline days (days that subjects had to be free of AMD diagnosis), study days (days after completion of baseline, during which subjects were interrogated for occurrence of AMD) and days on dabigatran.

**Table 2:** Statistical analysis of AMD and dabigatran use.

Years of exposure to dabigatran	Mean (SD) days of dabigatran	% patients with an AMD diagnosis	% ADM per study year	% patients with nAMD diagnosis	% nADM per study year	mean (SD) days in study
none	0	13.95*	4.67*	2.29*	0.68*	1090 (616)
dabigatran	398 (327)	9.10	3.07	0.91	0.28	1080 (620)
dabigatran:						
< 1 year	153 (105)	7.51	3.04	0.75	0.28	900 (610)
1-2 years	532 (94)	10.11	3.25	0.90	0.26	1135 (549)
2+ years	903 (132)	12.26	3.00	1.35	0.29	1492 (491)

Subjects were subdivided into dabigatran use or none, as well as stratified based on years of exposure to the drug during the study period. Patients who received dabigatran had a lower incidence of AMD, and developed less nAMD per study year than control subjects *p <0.0001 when compared to dabigatran across all exposure times, irrespective of the number of years on the drug.

### Dabigatran Reduces CNV Lesion Size and Accelerates Repair

The mouse CNV model is an accepted model for wet AMD, and is characterize by two phases, an injury and angiogenesis phase followed by slow repair. Giani and coworkers have shown that in OCT images, maximum CNV size is observed 5 days after the laser burn, followed by a slow repair (40). We have added to this observation, demonstrating that inhibiting the alternative pathway of complement accelerated fibrotic scar resolution, but that repair required homeostatic levels of the anaphylatoxins C3a and C5a (23).

Here we asked whether dabigatran would alter the course of angiogenesis or repair, by feeding animals with dabigatran chow during the two different phases. Based on chow provided by Boehringer Ingelheim, animals were dosed with dabigatran at ~10 mg/kg bodyweight per day, which according to Pingel and coworkers, using the identical chow, results in a blood plasma concentration of dabigatran (0.372 ± 0.03 µg/ml) and elongated the coagulation times by 3-fold (41). This level of dabigatran resulted in thrombin activity based on fluorogenic thrombin specific peptide cleavage (39) that was reduced by ~35% in serum (control: 100 ± 7.6; dabigatran: 65.4 ± 5.1; *P*=0.003), and ~10% in retina/RPE/choroid tissues (control: 100 ± 3.1; dabigatran: 91.1 ± 2.6; *P*=0.04) when compared to control mice.

**Figure 1:**
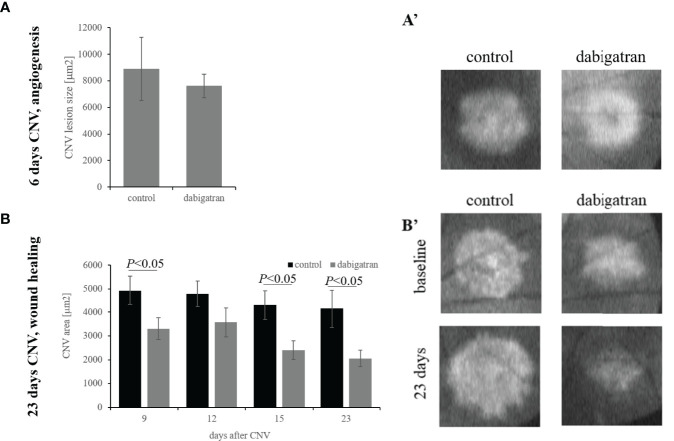
Dabigatran does not reduce CNV lesion size but does accelerate repair. Argon laser photocoagulation of Bruch’s membrane was used to generate four laser spots in each eye of the C57BL/6J mice. Mice were analyzed by OCT on day 5 post laser **(A)** or on days 5, 9, 12, 15 and 23 during the repair paradigm **(B)**. Mice were fed standard or dabigatran chow (~10 mg/kg bodyweight per day) during either the induction phase of the CNV lesion or during the repair phase, starting at day 5. Quantification of the lesion areas using image J analysis on OCT images demonstrate no reduction in CNV size in the short-term paradigm, and an acceleration of repair in dabigatran-fed mice. Data shown are average values ( ± SEM) per lesion and corresponding representative images of CNV lesions are shown.

Dabigatran, when provided from 2 days prior to CNV induction through the angiogenesis phase, resulted in a small and insignificant reduction of CNV lesion sizes by ~15% (*P*= 0.2) (**Figure 1A**). In contrast, when dabigatran was provided from day 5 after CNV induction through the repair phase, when analyzing lesion sizes at the final time point on day 23 using *post-hoc* testing (Fisher’s PLSD; StatView, SAS Institute), dabigatran-treated animals had significantly smaller lesions (*P*<  0.02). Since the same animals were followed over a 23-day time course, the treatment responses over time were evaluated using a repeated measure ANOVA. In the analysis of response over time, there was a significant effect of treatment (*P*< 0.0001), which was confirmed by Bonferroni (*P*< 0.0001) (**Figure 1B**).

### Thrombin Reduces Barrier Function in RPE Cells and Induces CTGF Expression and VEGF Secretion

**Figure 2:**
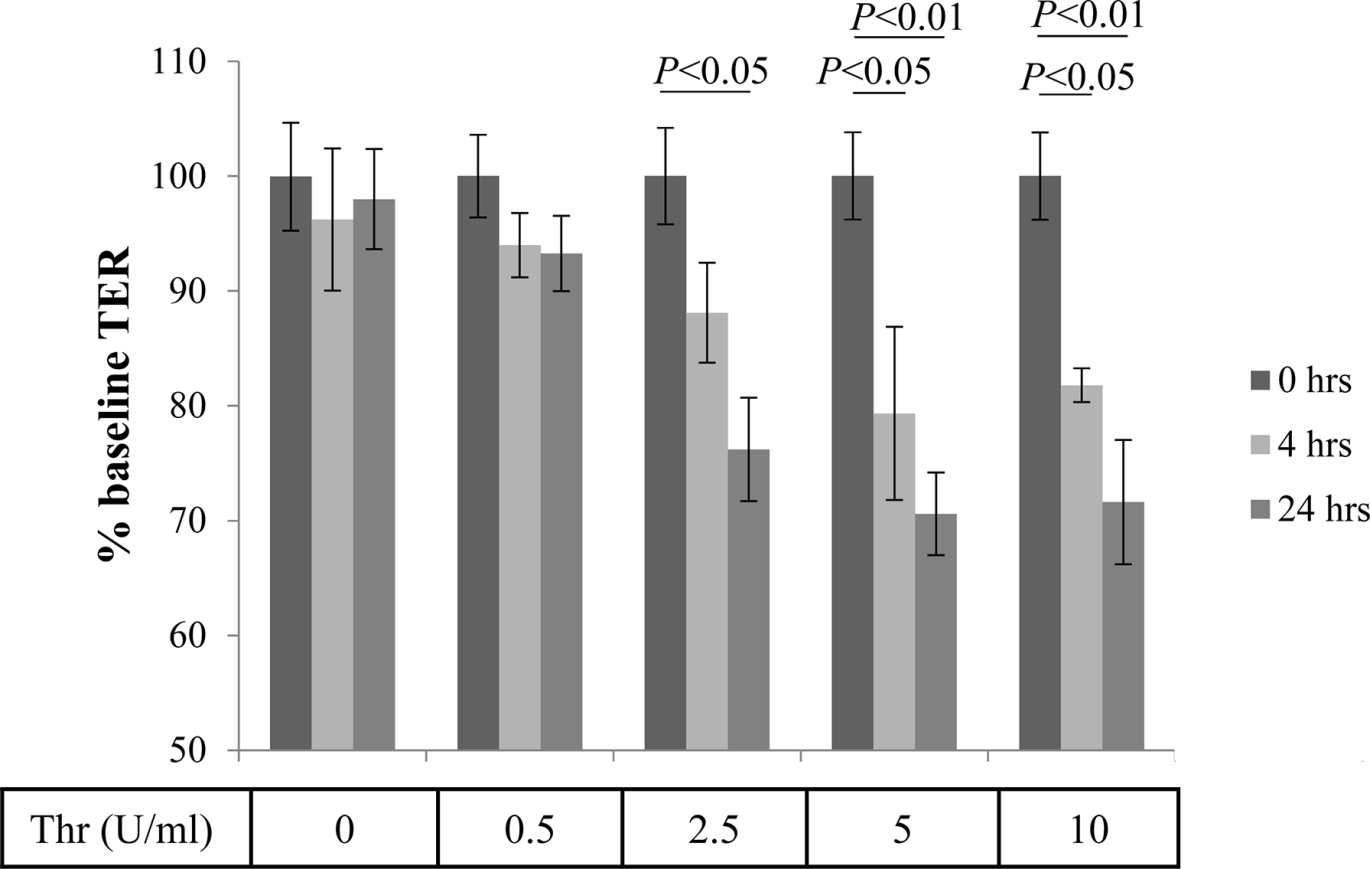
Thrombin-mediated effects of Transepithelial Resistance (TER). Stable monolayers of ARPE-19 cells grown on transwell plates were treated with increasing concentrations of thrombin (in U/mL), which resulted in a time and dose-dependent reduction of transepithelial resistance (TER). Data are plotted as mean ± SEM; n=3 independent experiments.

ARPE-19 cells are a good model to test for pathways involved in establishment and loss of barrier function (30), and transepithelial resistance (TER) has been found to correlate with VEGF secretion (34). Thrombin is a serine-protease and is a known contributor to the damage of the blood-brain barrier (16). To examine the effects of thrombin on the blood-retina barrier (i.e., TER), we performed dose- (0.5 -10.0 U/ml) and time- (4 and 24 hrs) dependent treatments on stable ARPE-19 cell monolayers, and compared treatment effects on TER levels to TER at baseline (serum free media/SFM before treatment, 0 hrs) (**Figure 2**). TER was stable over the 24 hr time course in untreated cells (0 U/mL), and was barely affected by the lowest dose tested (0.5 U/mL). Thrombin at a low concentration (2.5 U/ml) required a longer period of time (24 hrs) to cause significant damage to the epithelial barrier integrity. In contrast, higher concentrations of thrombin (5 U/ml and 10 U/ml) caused significant barrier function loss at the earlier time point (4 hrs).

**Figure 3:**
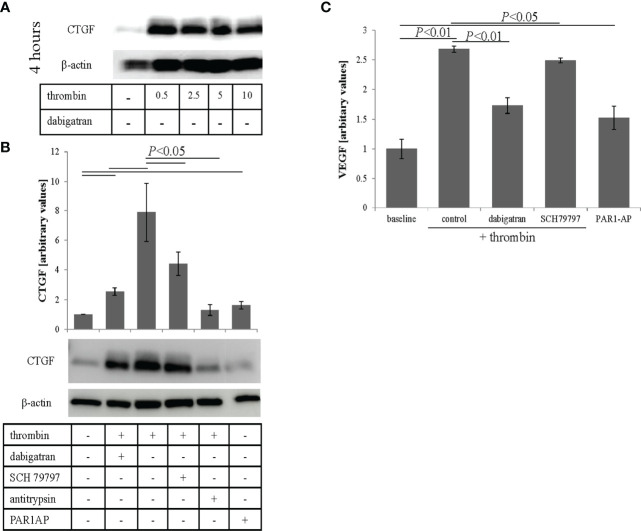
Thrombin-mediated effects on CTGF and VEGF expression. **(A)** Cell lysates were analyzed for thrombin-mediated CTGF expression. Thrombin induced expression of CTGF, that was already maximal at 0.5 U/mL. **(B)** Densitometric analysis demonstrated that thrombin-induced CTGF expression could be reduced by dabigatran (10 μM), the protease activated receptor-1 (PAR1) receptor antagonist (SCH79797; 250 μM), and the non-specific protease inhibitor (α1-antitrypsin; 1 mg/mL), and could be triggered using PAR1-AP (30 μM), a PAR1 agonist. **(C)** VEGF secretion (assessed by ELISA) into the apical supernatant could be induced by thrombin treatment and reduced by dabigatran and the selective non-peptide PAR1 receptor antagonist SCH79797. VEGF secretion into the apical supernatant could be triggered using the PAR1-AP. Data are plotted as mean ± SEM; n = 3 independent experiments.

Oxidative stress has been shown to lead to VEGF secretion in ARPE-19 cells (34), which in wet AMD is associated with abnormal blood vessel formation (42). As thrombin has been shown to induce CTGF expression (43) and CTGF is essential in the production of VEGF (44), we investigated whether thrombin has similar effects in RPE cells. ARPE-19 cell monolayers were treated with thrombin (0.5–10 U/ml) and cell lysates were collected and analyzed for CTGF levels at 4 hrs post treatment by western blotting. Thrombin was found to increase CTGF expression that however did not appear dose-dependent (**Figure 3A**). As lower concentrations optimally activated CTGF expression (0.5-2.5 U/mL) and dabigatran (10 μM) is less effective at reducing thrombin activity at higher concentrations (see **Supplementary Figure 1A**), the remaining experiments were performed at 1 U/mL. Thrombin can exert an effect *via* the thrombin receptor, Protease Activated Receptor-1 (PAR1). This direct thrombin pathway was investigated in cells treated with thrombin in the presence of dabigatran (10 μM), the PAR1 antagonist (SCH79797, 250 μM), the general protease inhibitor alpha1-antitrypsin (1 mg/mL) and the PAR1 activating peptide PAR1-AP (30 μM) (**Figure 3B**). Densitometric analyses of the blots from three independent experiments showed that thrombin significantly increased CTGF expression (*P*< 0.05), an effect that was blunted by dabigatran (3.2 fold) and alpha1-antitrypsin (5.3 fold). Specificity for PAR1 was provided by utilizing the PAR1 antagonist SCH79797 (1.7 fold), which partially reduced the expression of CTGF and the PAR1 agonist PAR1-AP which marginally increased the expression (1.5 fold). To investigate whether the levels of CTGF are correlated with VEGF secretion in thrombin-treated ARPE-19 cell monolayers, the apical supernatants from treated monolayers were used for ELISA assays. Thrombin alone resulted in a 2.5-fold increase of VEGF in APRE-19 cell apical supernatants, an effect that was partially blocked by dabigatran, reducing the VEGF content by ~35% (**Figure 3C**). Treatment with the PAR1 agonist increased the secretion of VEGF. Overall, the results suggest that thrombin reduces barrier function in RPE cells in part by stimulating VEGF secretion, an effect that might be mediated in part by PAR1-mediated CTGF expression.

### Cross-Talk Between the Complement and Coagulation System in ARPE-19 Cell Monolayers

Here, a second mechanism was investigated. We have previously shown that complement activation on RPE cells impairs barrier function requiring sublytic membrane attack complex (MAC) activation and VEGF secretion (33), and thrombin has been reported to be able to cleave complement components (17, 18). As a prerequisite for the experiments described here, it was confirmed that dabigatran inhibits thrombin activity in a cell-free system, whereas the two complement inhibitors used here, compstatin and TT30, did not (**Supplementary Figure 1B**).

**Figure 4:**
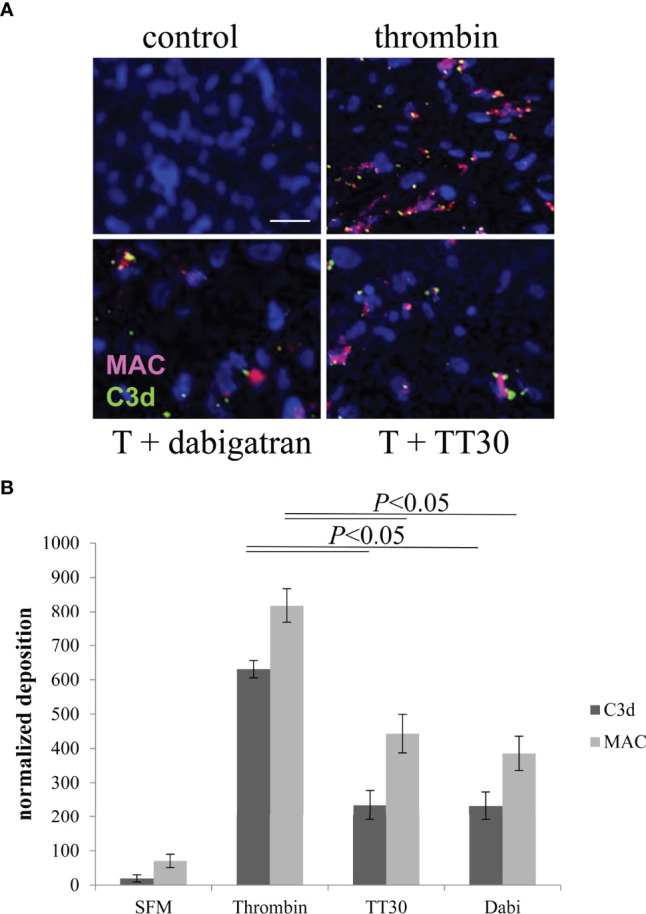
Thrombin-mediated effects on cell-surface activated complement. **(A)** ARPE-19 cells plated on glass-bottom dishes were assessed for complement activation based on C3d (green) and MAC (red) deposition on the apical surface. Cell bodies were identified using Hoechst. ARPE cells were either treated with serum-free media (control) or with 20 U/mL of thrombin in the presence and absence of dabigatran (10 μM) or TT30 (10 μM). Please note that ARPE-19 cells secrete complement proteins into the supernatant, sufficient to mount a complement attack. **(B)** C3d and MAC deposits counted by image J were significantly were identified in thrombin treated cells and reduced significantly in cells treated with dabigatran or TT30. Data are plotted as mean ± SEM; n = 3 independent experiments.

First, we tested whether thrombin results in deposition of C3d and the assembly of MAC on ARPE-19 cell surfaces. ARPE-19 cell monolayers were treated apically with thrombin and deposition of MAC (C5b-9; red) and C3d (green) were examined by immunofluorescence staining (**Figure 4A**). Thrombin treatment induced both C5b-9 and C3d accumulation compared to control (serum free medium; SFM) (**Figure 4A**). Both C5b-9 and C3d deposition were reduced with pretreatment of dabigatran (10 μM) (**Figure 4B**), or with the alternative pathway of complement inhibitor TT30 (10 μM) (33) (**Figure 4B**). Image analysis showed that dabigatran and TT30 both reduced C3d deposition by ~60% when compared with thrombin-treated cells. Similarly, C5b-9 deposition was reduced by ~50% by either of the two inhibitors when compared with thrombin-treated cells. As TT30 inhibits the AP pathway on membranes, this suggests that thrombin activation results in C3b deposition on cell membranes that is amplified by the AP.

**Figure 5:**
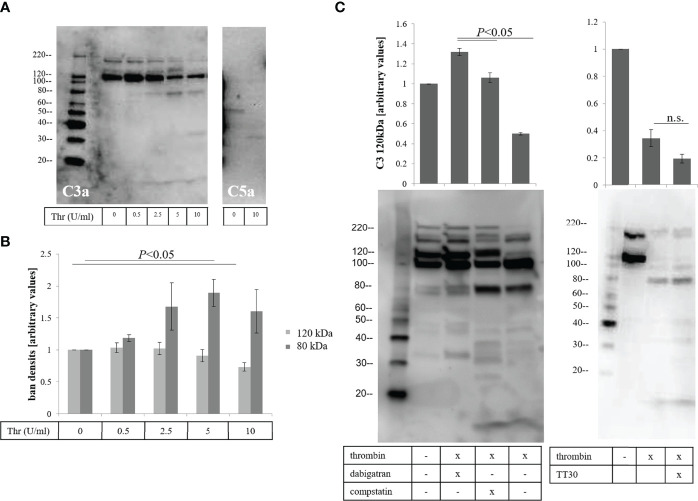
Thrombin-mediated effects on fluid phase-dependent complement activation. **(A)** Apical supernatants of treated cells were analyzed for thrombin-mediated complement component C3 (left blot) and C5 (right-hand blot) cleavage, using antibodies against C3a and C5a respectively. **(B)** Reduction of the 120 kDa and generation of an 80 kDa fragment of C3 are assessed by densitometric analysis using image J. **(C)** Reduction of the 120 kDa band could be inhibited by dabigatran (10 μM) and the fluid phase complement inhibitor compstatin (100 μM) (left hand blot) but not the inhibitor of the alternative pathway TT30 (10 μM) that requires membrane binding (right hand blot). Data are plotted as mean ± SEM; n = 3 independent experiments.

Second, we asked whether thrombin can cleave complement components (17, 18), specifically C3 and C5, producing cleavage products potentially different from those produced by classicall convertases. To test the effects of thrombin on complement cleavage, the apical supernatants of treated ARPE-19 cell monolayers were analyzed by western blotting. Cleavage of C3α was evaluated using an antibody specific for C3a (**Figure 5A**, left-hand blot). The C3a antibody detected C3, the C3α band as well as other fragments containing C3a. Thrombin caused a concentration dependent cleavage of complement component C3 (~190 kDa), caused reduction of the 120 kDa C3α band and generation of a new 80 kDa fragment. We also observed fragments at 32 kDa and 9-10 kDa with higher doses of thrombin treatment (**Figure 5A**, 10 U/mL). The 9-10 kDa band ran at the same molecular weight as purified C3a (**Supplementary Figure 2**), but whether the thrombin-produced fragment is the same as the C3-convertase generated C3a requires peptide sequencing. Likewise, the identify and cleavage site of in C3α to generate the 80 kDa band was not further investigated here. The reduction of the 120 kDa fragment was correlated with the generation of the 80 kDa fragment (**Figure 5B**). The loss of the 120 kDa fragment was prevented by either dabigatran (10 μM) or the C3 inhibitor compstatin (100 μM), which inhibits complement both on cell membranes as well as in fluid phase, but not by TT30 (10 μM), which is an inhibitor that prevents membrane-bound AP activation (**Figure 5C**). In addition, thrombin on cleavage of complement component C5α could be demonstrated using an antibody against C5a (**Figure 5A**, right-hand blot). We observed a unique 35 kDa, C5a-like fragment as previously shown by Krisinger (18).

**Figure 6:**
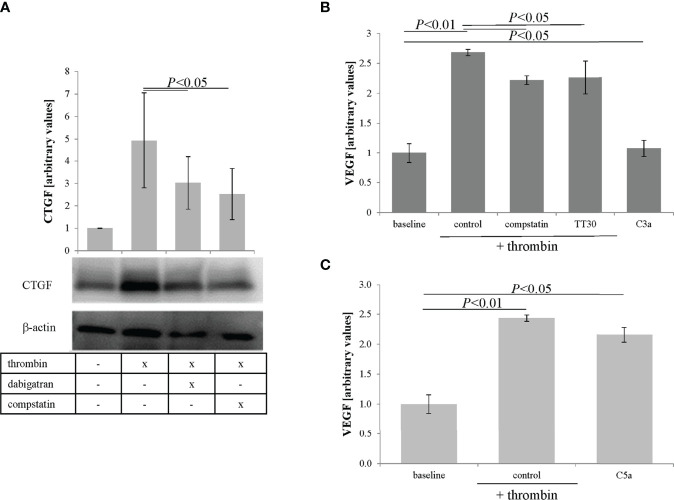
CTGF and VEGF expression can be regulated by complement. **(A)** Thrombin-mediated CTGF expression in cells can be inhibited by fluid phase complement inhibition (compstatin). **(B)** VEGF secretion into the apical supernatant as measured by ELISA was induced by thrombin, partially reduced by fluid phase or membrane-based complement inhibition (100 μM) and partially induced by C3a receptor activation (C3a, (260 nM). **(C)** C5a receptor stimulation (C5a, 52 nM) was as potent as thrombin treatment when assessing the amount of VEGF secreted into the apical supernatant. Data are plotted as mean ± SEM; n = 3 independent experiments.

Finally, to tie in CTGF and VEGF, CTGF expression and VEGF secretion were analyzed in response to thrombin and compstatin. Inhibition of complement by compstatin was found to partially reduce thrombin-induced CTGF expression (**Figure 6A**) and VEGF secretion (**Figure 6B**). Interestingly, a significant driver of VEGF secretion was found to be C5a, increasing VEGF levels to that identified by thrombin stimulation (**Figure 6C**), whereas C3a was a weak stimulator. Overall, the data suggests that thrombin-mediated CTGF expression is partially regulated by complement activation; and VEGF secretion appears to be triggered by thrombin in a combined fashion, involving PAR1 activation, presumably sublytic MAC activation based on the observed MAC deposition and our previous data (33), and C5a receptor activation. It will be of great interest to further investigate the activity of the 32 kDa C5a-containing cleavage fragment.

### Pathway Analysis in CNV

Based on the results in cells, we followed up the animal analysis, focusing on VEGF levels in the RPE/choroid, complemented by CTGF analysis and complement activation (C3d production and binding). RPE/choroid samples were collected at the end of the angiogenesis experiment (day 6) and after the final OCT analysis in the repair experiment (day 23) and compared to naive controls (no CNV and no dabigatran treatment).

**Figure 7:**
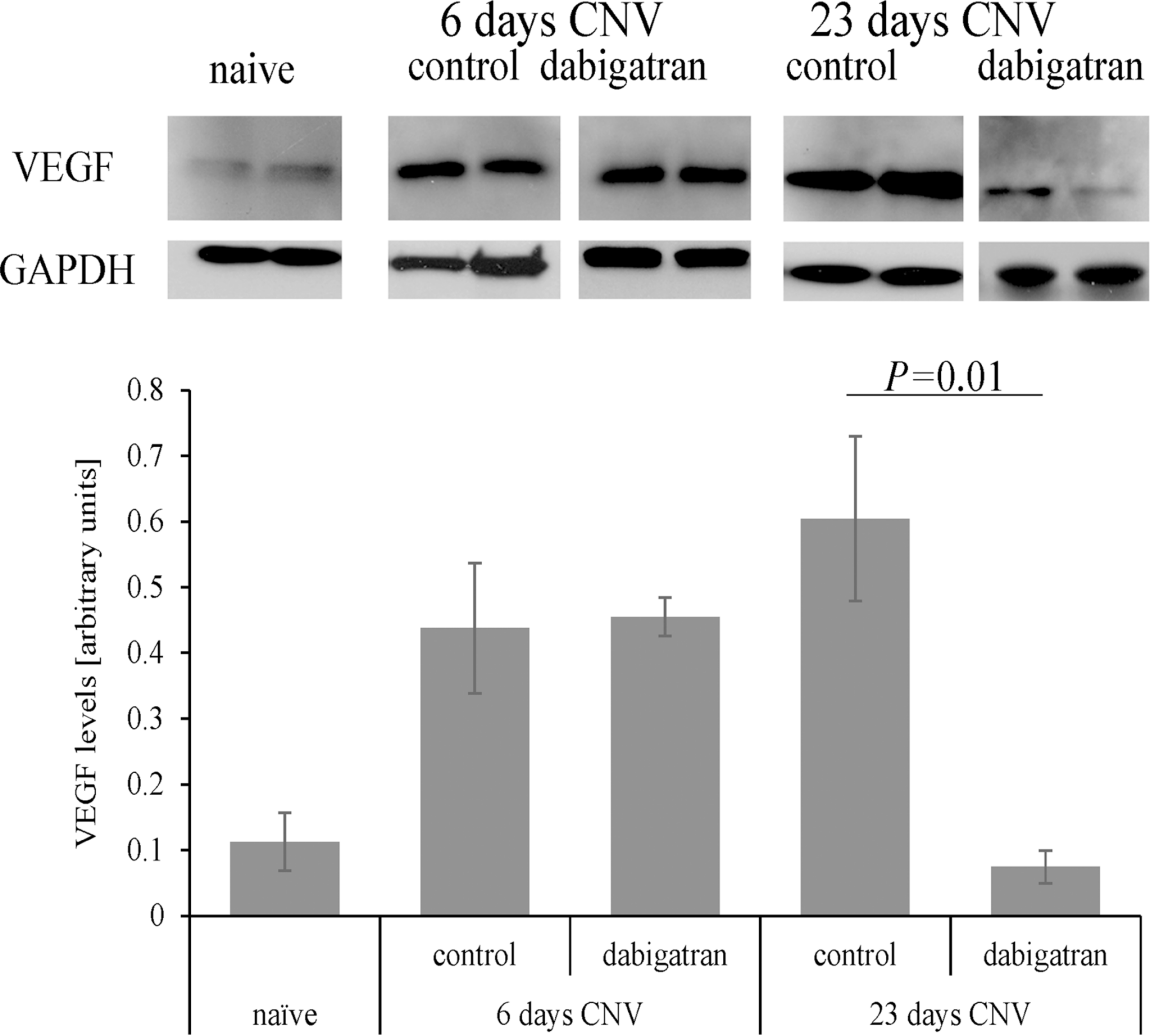
Dabigatran affects VEGF expression in repair but not angiogenesis phase of CNV. Equal amounts of RPE/choroid extracts (15 μg/lane) were loaded per lane, probed with antibodies as indicated, and band intensities quantified. Arbitrary values were established based on normalization with GAPDH. Age-matched naive animals without CNV and fed normal mouse chow were compared to those in which CNV was and treated with dabigatran during the angiogenesis and repair phase of CNV. During the induction phase and repair phase of CNV, VEGF levels in the RPE/choroid were elevated; however, dabigatran only affected VEGF levels in the long-term paradigm.

As expected, VEGF levels were elevated in response to CNV in PBS treated animals 6 days after CNV induction (*P*=0.03), and remained elevated at the 23-day time point (*P*=0.01) (**Figure 7**). And while dabigatran had no effect on VEGF levels at the 6-day time point (*P*=0.9), dabigatran significantly reduced VEGF levels at the 23-day time point (*P*=0.001). Dabigatran’s effect on VEGF levels were correlated with its effect, or lack thereof, on the size of the CNV lesions at 23 and 6 days, respectively.

**Figure 8:**
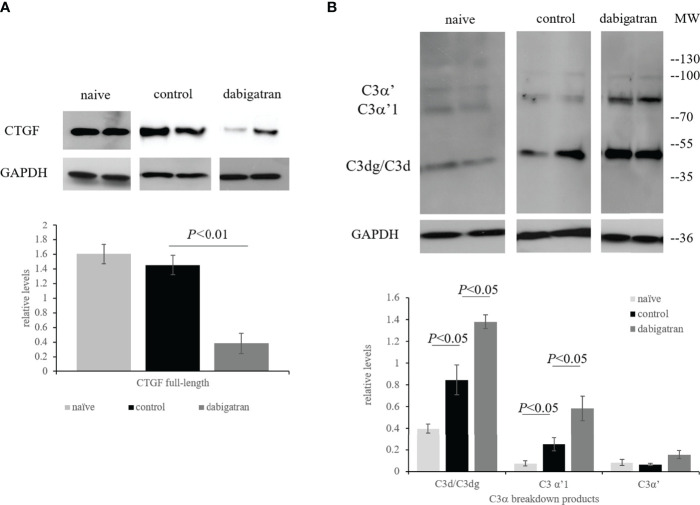
In repair phase of CNV, dabigatran reduces CTGF expression. Western blot analysis was performed as described in legend for **Figure 7**. **(A)** Dabigatran reduced CTGF in the wound healing paradigm when compared to the control-treated and naïve animals. **(B)** Complement activation was increased in RPE/choroid of CNV animals when compared to naïve animals. Surprisingly, complement activation was further increased by treatment when compared to PBS controls. Data are expressed as mean ± SEM (n = 3-5 independent samples per condition).

Based on the observed effects of dabigatran on the wound healing component of CNV, these were the only samples that were followed by further. In all three groups the full-length 38 kDa band of CTGF was present (**Figure 8A**). At the end of the repair period (23 days), levels were indistinguishable between naïve and control food CNV animals, whereas dabigatran significantly reduced the levels of CTGF (*P*< 0.01).

Unexpectedly, and in contrast to the cell data (**Figure 4**), C3d deposition was not reduced by dabigatran. As shown previously, when examining C3α breakdown using our C3d antibody (group 2, clone 11) which recognizes the C3d epitope present in the C3α chain and its breakdown fragments, they can be distinguished according to their molecular weights (38). At the end of the repair period (23 days), the C3α breakdown products C3d/C3dg and C3α’1 continue to be elevated with CNV (*P*< 0.05), and both levels are further elevated by dabigatran (*P*< 0.05) (**Figure 8B**). Overall, the data suggests that in CNV, thrombin-mediated effects could be identified in the wound healing phase, but not during angiogenesis. Dabigatran was found to reduce CTGF and VEGF expression levels in CNV that was correlated with its effect on CNV lesion sizes. Surprisingly, and in opposition to the results identified in ARPE-19 cell cultures, inhibition of thrombin by dabigatran was found to increase the presence of complement breakdown products in RPE/choroid of CNV lesioned animals. It will be of great interest to further investigate the effects of dabigatran on other aspects of angiogenesis, fibrosis and wound healing.

## Discussion

**Figure 9:**
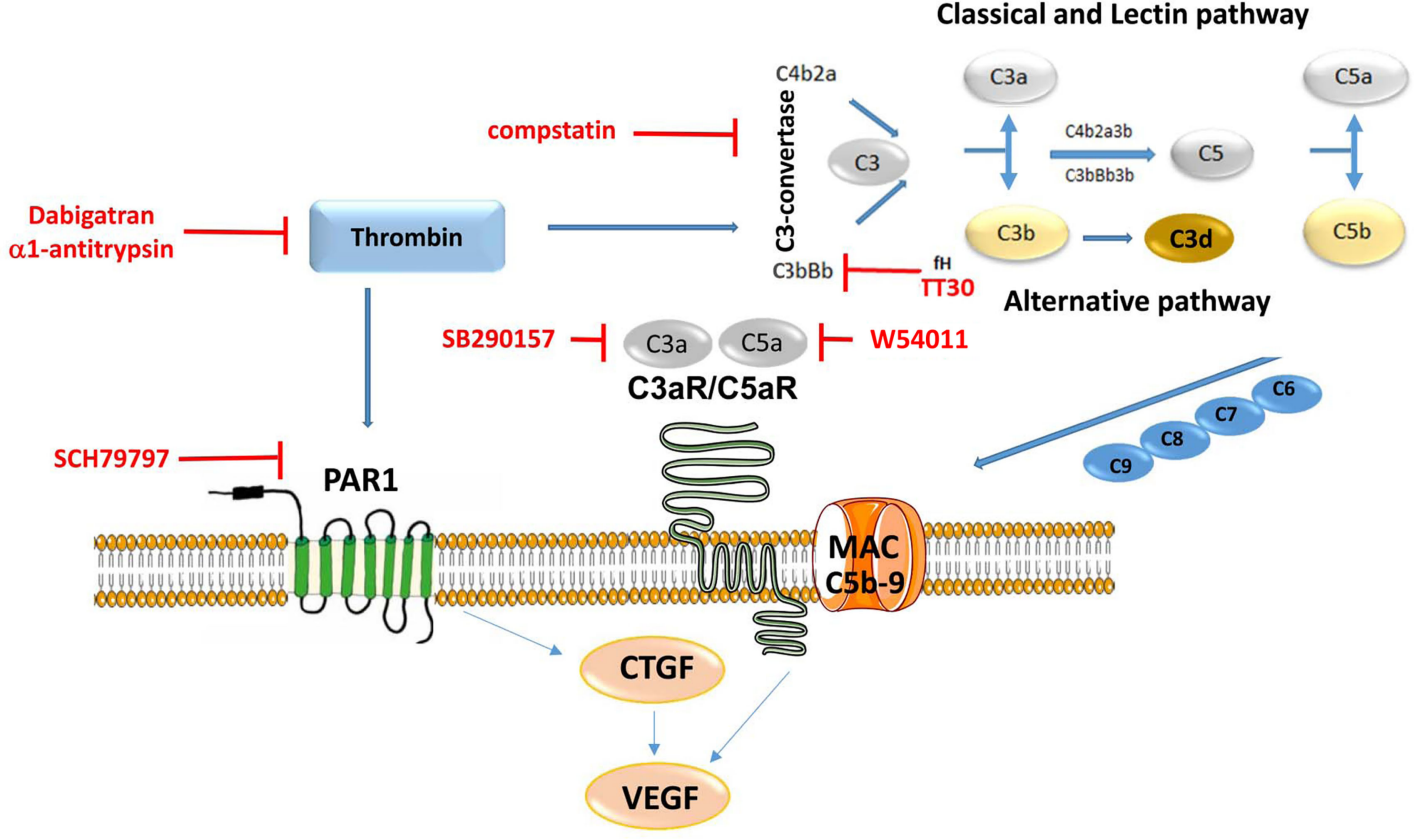
Summary Figure. Thrombin regulates a dual-signaling mechanisms, by cleaving C3 and C5, it activates the complement cascade, and it modulates down-stream signaling *via* membrane-bound receptor PAR1. Inhibitors are presented in red and pathways are presented in blue. The study concluded that thrombin induces complement and CTGF, which subsequently activates VEGF secretion in ARPE-19 cells, resulting in disassembly of tight junctions and TER reduction.

The coagulation and the complement system are two systems, in which activation leads to the assembly of proteolytic complexes, and their activities are tightly regulated by a set of specific activators and inhibitors. The proteolytic complexes are made up mainly of serine proteases that exhibit high substrate specificity. The crosstalk between these two systems has been studied in many diseases, with physiological consequences including the regulation of the immune system (45–47). The contributions of complement activation to pathology have been established in AMD, however, little is known about the coagulation system in this disease, or the potential interrelationship between coagulation factors and complement components. This study was focused on exploring the potential role of thrombin in AMD. The overall findings of this study are as follows: 1) dabigatran use reduces the risk for AMD, and in particular wet AMD in carefully matched Medicare populations, 2) thrombin induces its effects on RPE cell physiology *via* a dual mechanism, 3) thrombin cleaves complement component C3, C5 and activates the terminal complement pathway, leading to C3d and MAC deposition, 4) thrombin triggers both PAR1 receptor and complement-mediated CTGF production, 5) thrombin induces VEGF secretion *via* both *via* PAR1 receptor activation and complement activation, 6) dabigatran accelerates CNV lesion repair, 7) and modifying effects of dabigatran on CTGF and VEGF expression could be verified, but not of complement activation. Overall, a therapeutic effect of dabigatran could be identified in patients, a mouse model of disease and a cell-based model. However, the hypothesis of a potential crosstalk between thrombin and complement components could only be verified in the cell model (**Figure 9**).

Thrombin is a zymogen, activated by coagulation factor X by proteolytic cleavages at Arg271 and Arg320 in a process of blood coagulation system activation (48). Association of dysregulated thrombin activation has been demonstrated in proliferative vitreoretinopathy (PVR), which is also a VEGF- and complement-associated (49, 50) ocular disease. In a late-stage, case-controlled study on AMD, levels of prothrombin fragment F1.2 (F1.2; a molecular marker of thrombin generation *in vivo*) was marginally lower in AMD compared with controls (51). Dabigatran is a selective direct thrombin inhibitor and is therefore prescribed as an anticoagulant. It’s mechanism of action is summarized by Dong and colleagues (52), and includes reversibly binding to the active site of thrombin, preventing fibrinogen cleavage and thus the formation of insoluble fibrin. Dabigatran bound to thrombin prevents an essential cleavage step of the extracellular N-terminal of the PAR1 receptor required for signaling (53), inhibiting many thrombin-induced profibrotic events, including collagen production by lung fibroblasts. Finally, thrombin activity has been shown to be involved in angiogenesis (54), modulating VEGF secretion (55).

Using the MarketScan database, we were able to examine a large number of carefully matched patients to examine the hypothesis that reducing thrombin activity by dabigatran reduces the risk of AMD. Here we report that risk of AMD diagnosis and in particular wet AMD diagnosis is reduced in patients with prescriptions for dabigatran compared to a matched control group. Both the total rate of all new AMD cases and the rate of wet AMD events were reduced, independent of the years of exposure to the drug during the study period. It would be of great interest to determine whether the severity of the disease is also affected by dabigatran exposure, i.e, whether fewer anti-VEGF treatments are needed to control the disease or whether treat and extend intervals can be prolonged.

To understand mechanisms of thrombin-mediated changes in one of the cell types affected in AMD, we used ARPE-19 cells grown as stable monolayers on transwell plates, that we have used previously to analyze complement activation in response to oxidative stress (33), smoke exposure (35), as well as the complement pathway involved (56). Here we added to this analysis examining the effects of thrombin activation as well as the cross-talk between thrombin and the complement system. RPE cells have been shown to express thrombin PAR1 and PAR3 receptors; however, the predominant form appears to be the PAR1 receptor (57, 58). The basal side of the RPE is naturally exposed to blood-derived compounds and is expected to be protected, whereas the apical side is protected due to the barrier function of the RPE. However, upon destruction of the BRB in disease, both complement and thrombin can get access to the apical side. In initial experiments we compared the effects of thrombin when added to the basal or the apical side of the RPE. TER measurements showed that thrombin caused reduction of TER only when added to the apical side but not the basal side (data not shown). In addition, no thrombin-mediated C3α cleavage products could be identified in the basal supernatant (data not shown). Our approach was similar to that of other groups that have tested the effects of thrombin on RPE cells when added to the apical side, such as the effects of thrombin on glutamate release (59) or cytokine secretion (55) among others, but is in contrast to human colon-derived epithelial cells, in which changes in barrier function loss could be induced equally by incubation with either apical or basolateral PAR1 agonists (60).

The cell-based study suggested that thrombin, *via* activation of PAR1 receptor signaling resulted in an increase in CTGF and VEGF expression. Interestingly, higher thrombin activity is present in vitreous fluid collected from PVR patient, and that PVR vitreous induces expression of cytokines/chemokines and growth factors in ARPE-19 cells (61), with many of these cytokines, chemokines, and growth factors facilitating the generation of a microenvironment for neovascularization in disease models (62–66). In primary human RPE cells dose- and time-dependent thrombin treatment was shown to induce VEGF-secretion *via* PAR1 receptor activation (55). One of the conclusions from this study is that thrombin does not trigger a unidirectional cell-signaling mechanism, but rather triggers multiple secondary messengers and works in combination with cytokines and growth factors (55). This might also explain why blocking complement or thrombin receptors alone did not completely block CTGF or VEGF expression and secretion. To reduce thrombin-mediated RPE pathology may therefore require a multipronged approach.

One of the major findings of the cell-based study is that thrombin can lead to the deposition of MAC on RPE cells as well as activate the complement cascade by cleaving complement component C3 and C5 into novel breakdown products, and therefore may be able to substitute for the requirement of C3/C5-convertase enzymes in RPE cells. In general, complement cascade activated by 3 pathways: the classical pathway (CP), the lectin pathway (LP), and the alternative pathway (AP). All pathways lead to activation of a series of serine proteases (C3 and C5 convertases), generate anaphylatoxins, opsonize the non-self/target cells, initiate MAC formation, and eventually lysed the cells (67, 68). In AMD, the AP appears to be the major contributor of complement activation leading to pathogenesis. Here we showed that thrombin activates the complement cascade in a non-conventional way, generating C3 and C5 breakdown products. The C3α breakdown products include a new 80 kDa fragment, a 32 kDa fragment and 9 kDa band that run at the same molecular weight as purified C3a. In the absence of sequence analysis of the C3a-like protein, it is unclear whether true C3a is generated; and since the measured effect of thrombin was not mediated by C3a (thrombin mediated effects on VEGF could not be mimicked by C3a or blocked in combination with a C3a-receptor antagonist), this question cannot be answered. The C5α breakdown product included a new 35 kDa fragment that could be identified with a C5a antibody. The cleavage site was previously identified by Krisinger and colleagues as the highly conserved thrombin-sensitive R947 site (18). Interestingly, in their study, they showed that the truncated C5b [C5b(T)] generated by thrombin in the context of a functioning complement system, was able to form a C5b(T)-9 MAC pore that was significantly more lytic than regular C5b-9 MAC (18). Support of the hypothesis that the 32 kDa C5a-containing fragment is bioactive comes from data by the Huber-Lang laboratory who showed using ELISA that human C5 incubated with thrombin results in the generation of C5a or C5a-containing fragments, as the reaction products were able to induce chemotaxis in human neutrophils (17). However, thrombin not only activates complement signaling, but also its inhibition by upregulating decay accelerating factor (DAF, CD45) expression on endothelial cells (69). Wiegner and coworkers have summarized our understanding of the “crosstalk” between components of the complement and the coagulation system. Importantly, components of the coagulation cascade can cleave and/or activate proteins of the complement system in fluid phase, but proteins from the two cascades can interact on many different surfaces such as endothelial cell membranes (stationary surfaces) as well as circulating entities (70). Our data on thrombin-mediated C3 cleavage (inhibited by compstatin, but not TT30) and MAC deposition (inhibited by TT30) as well as thrombin-mediated VEGF secretion (partially inhibited by compstatin and TT30), provide evidence for both mechanisms in ARPE-19 cells. Overall, our data suggests that thrombin should be considered consider in the context of drug development to control aberrant complement activation.

In the mouse model of CNV, dabigatran was found to accelerate the resolution of the fibrotic scar. The fibrotic scar size over time was reduced by dabigatran, and was associated with a reduction in CTGF, and the amount of CTGF was highly correlated with the expression levels of VEGF (*P*< 0.0001). Interestingly, in the long-term scar resolution model, the CNV-induced increase in complement activation was not reduced, but rather significantly increased for the C3α breakdown products analyzed. Overall, the CNV model seems to support the findings in patients, that wet AMD risk is reduced by dabigatran use. The molecular analysis in the mouse model supports the findings in ARPE-19 cell monolayers that dabigatran reduces thrombin-induced CFGF expression and thereby reduces the amount of VEGF. However, the hypothesis that dabigatran might expose the presence of a cross-talk between the complement and the coagulation system was not supported. Additional experiments are required to tackle this question.

In the eye, complement homeostasis is influenced by both levels of systemic circulating complements and ocular complement production (71). To regulate an overactive AP pathway in the RPE/BrM/choriocapillaris, the main targets of complement activation in AMD impose unique challenges. Using systemic delivery of complement inhibitors might be limited by BrM, which restricts access for larger and glycosylated proteins (72), whereas intravitreal administration of drug in the context of a healthy or partially damaged BRB may show limited transport of therapeutics across the RPE due to a lack of appropriate transport systems (73). Additional challenges were also observed for anti-VEGF treatments, which are currently standard of care for patients with wet AMD. It has been shown that anti-VEGF responses are recipient-dependent, and therefore complicate dose optimization (74, 75). Many patients convert from intermediate, presumably complement dependent AMD to wet AMD, requiring anti-VEGF treatment, followed by conversion to geographic atrophy. To target each of the regulators (complement/VEGF) individually might complicate AMD treatment options further. Hence, the identification of a compound that acts upstream of both of these two pathways such as thrombin, will potentially open up a new method of treatment. Dabigatran etexilate is an approved oral drug, that is capable of lowering thrombin activity in systemic circulation and specific organs such as lung, heart and brain (76, 77), and might be a promising drug to lower thrombin-mediated outcomes in AMD.

## Data Availability Statement

The raw data supporting the conclusions of this article will be made available by the authors, without undue reservation.

## Ethics Statement

The animal study was reviewed and approved by The Institutional Animal Care and Use Committee (IACUC) at the Medical University of South Carolina.

## Author Contributions

TA designed, executed and analyzed the *in vitro* experiments and wrote part of the manuscript. BA analyzed the tissues from the mouse experiments. EO performed the mouse CNV *in vivo* experiments. KS performed the MarketScan analysis and interpreted the data. BR together with TA conceived the project, supervised the experiments, analyzed and interpreted the data and rewrote the manuscript. All authors contributed to the article and approved the submitted version.

## Funding

The study was supported by the National Institutes of Health (NIH) (EY030072, HS028284-01 and U66RH31458), the Department of Veterans Affairs (IK6BX004858, RX000444 and BX003050), the South Carolina Smart State Endowment, and the T32 Program in Immunology Research & Entrepreneurship at MUSC (T32AI132164).

## Conflict of Interest

The authors declare that the research was conducted in the absence of any commercial or financial relationships that could be construed as a potential conflict of interest.

## Publisher’s Note

All claims expressed in this article are solely those of the authors and do not necessarily represent those of their affiliated organizations, or those of the publisher, the editors and the reviewers. Any product that may be evaluated in this article, or claim that may be made by its manufacturer, is not guaranteed or endorsed by the publisher.

## Supplementary Material

Supplementary Figure 1 Thrombin activity is not affected by complement inhibitors. **(A)** Thrombin activity was assessed in a cell-free system. Cleavage of thrombin-specific substrate is proportionate to the concentration of thrombin (10 mU/ml -500 mU/ml) added, and could be blocked by the thrombin inhibitor dabigatran. **(B)** The alternative pathway inhibitor (TT30) that acts on cell membranes, and the C3-convertase blocker compstatin, which blocks complement both on membranes and in fluid-phase, both do not inhibit thrombin activity.

Supplementary Figure 2 Immunogenicity and molecular weight of the C3α fragment generated by thrombin mediated C3 cleavage. Supernatants of cells treated with thrombin, or TT30 were run in parallel with purified mouse C3a (Comptech). The low molecular weight bands produced in the presence of thrombin run at the same molecular weight as purified C3a.
