# Supplementary material for: Dabigatran and Wet AMD, Results From Retinal Pigment Epithelial Cell Monolayers, the Mouse Model of Choroidal Neovascularization, and Patients From the Medicare Data Base
Source: Front Immunol. 2022 Jun 17;13:896274. doi: 10.3389/fimmu.2022.896274 (PMC9248746; doi:10.3389/fimmu.2022.896274)
Supplement: Supplementary file 1 [file DataSheet_1.docx]

**Supplemental material**

**Supplemental methods**

**Thrombin activity in cell-free system**

To measure the level of active thrombin in cell-free system, known concentrations of thrombin were prepared in 100 µl of assay buffer (50 mM Tris, 100 mM NaCl, and 0.01% BSA; pH 7.5), subsequently 50 µl of thrombin substrate was added and incubated at 37°C. Fluorescence was measured at 405 nm using the Biotek Synergy HT Microplate Reader and thrombin activity was determined by extrapolation from a thrombin standard curve. Effects of inhibitors were tested by pre-incubating the inhibitors with thrombin, before substrate incorporation.

**Results S1**

**Complement inhibitors do not inhibits thrombin activity in a cell-free system.**

The goal is to investigate whether thrombin-mediated loss of barrier function is due to complement activation. To do so, we confirmed that thrombin activity is not affected by complement inhibitors TT30 and compstatin in a cell-free system. Dabigatran or complement inhibitors were incubated with thrombin at a range of concentrations (10 mU/ml to 100 mU/ml), and the end-point thrombin activity was estimated from fluorescence intensity of the reaction mixture. Thrombin was shown to cleave its substrate in a concentration dependent manner as expected (**Fig. S1**). While dabigatran (10 μM) was found to significantly reduce the enzyme activity over the entire range of thrombin concentrations (10-500 U/mL), it was most effective between 10-100 U/mL (**Fig. S1**). In contrast, complement inhibitors TT30 (10 μM) and compstatin (100 μM) did not affect thrombin’s enzymatic activity (Fig. S1B). No significant fluorescence signal reduction could be observed when using TT30 or compstatin, indicating these inhibitors do not interfere with thrombin activity.

**Figure S1: Thrombin activity is not affected by complement inhibitors.**

(**A**) Thrombin activity was assessed in a cell-free system. Cleavage of thrombin-specific substrate is proportionate to the concentration of thrombin (10 mU/ml -500 mU/ml) added, and could be blocked by the thrombin inhibitor dabigatran. (**B**) The alternative pathway inhibitor (TT30) that acts on cell membranes, and the C3-convertase blocker compstatin, which blocks complement both on membranes and in fluid-phase, both do not inhibit thrombin activity.

**Results S2**

**C3α fragment generated by thrombin mediated C3 cleavage.**

Cleavage of C3α was evaluated using an antibody specific for C3a. As a control for molecular weight and specificity of 9-10 kDa band detected by the C3a antibody, samples were run in the presence of purified mouse C3a (Comptech). The 9-10 kDa band ran at the same molecular weight as purified C3a, but whether the thrombin-produced fragment is the same as the C3-convertase generated C3a requires peptide sequencing.

**Figure S2: Immunogenicity and molecular weight of the C3α fragment generated by thrombin mediated C3 cleavage.**

Supernatants of cells treated with thrombin, or TT30 were run in parallel with purified mouse C3a (Comptech). The low molecular weight bands produced in the presence of thrombin run at the same molecular weight as purified C3a.
