# Supplementary figures and images for: Dabigatran and Wet AMD, Results From Retinal Pigment Epithelial Cell Monolayers, the Mouse Model of Choroidal Neovascularization, and Patients From the Medicare Data Base
Source: Front Immunol. 2022 Jun 17;13:896274. doi: 10.3389/fimmu.2022.896274 (PMC9248746; doi:10.3389/fimmu.2022.896274)

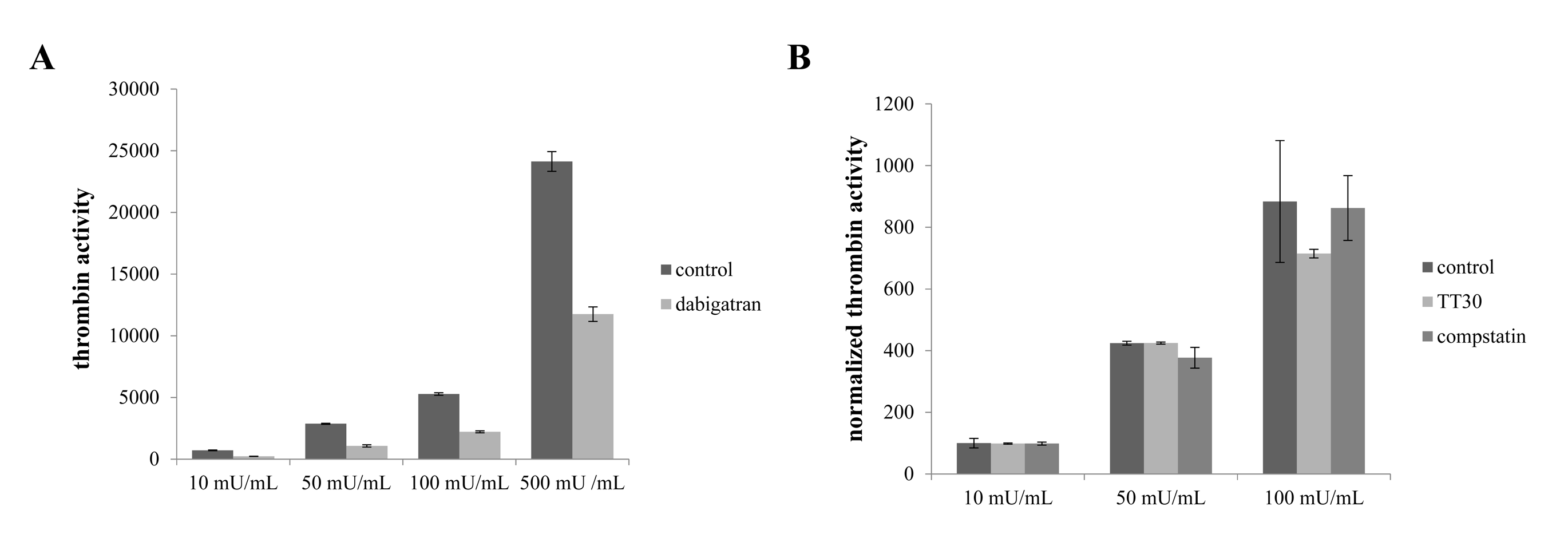

Supplement: Supplementary Figure 1 — Thrombin activity is not affected by complement inhibitors. (A) Thrombin activity was assessed in a cell-free system. Cleavage of thrombin-specific substrate is proportionate to the concentration of thrombin (10 mU/ml -500 mU/ml) added, and could be blocked by the thrombin inhibitor dabigatran. (B) The alternative pathway inhibitor (TT30) that acts on cell membranes, and the C3-convertase blocker compstatin, which blocks complement both on membranes and in fluid-phase, both do not inhibit thrombin activity. [file Image_1.tif]

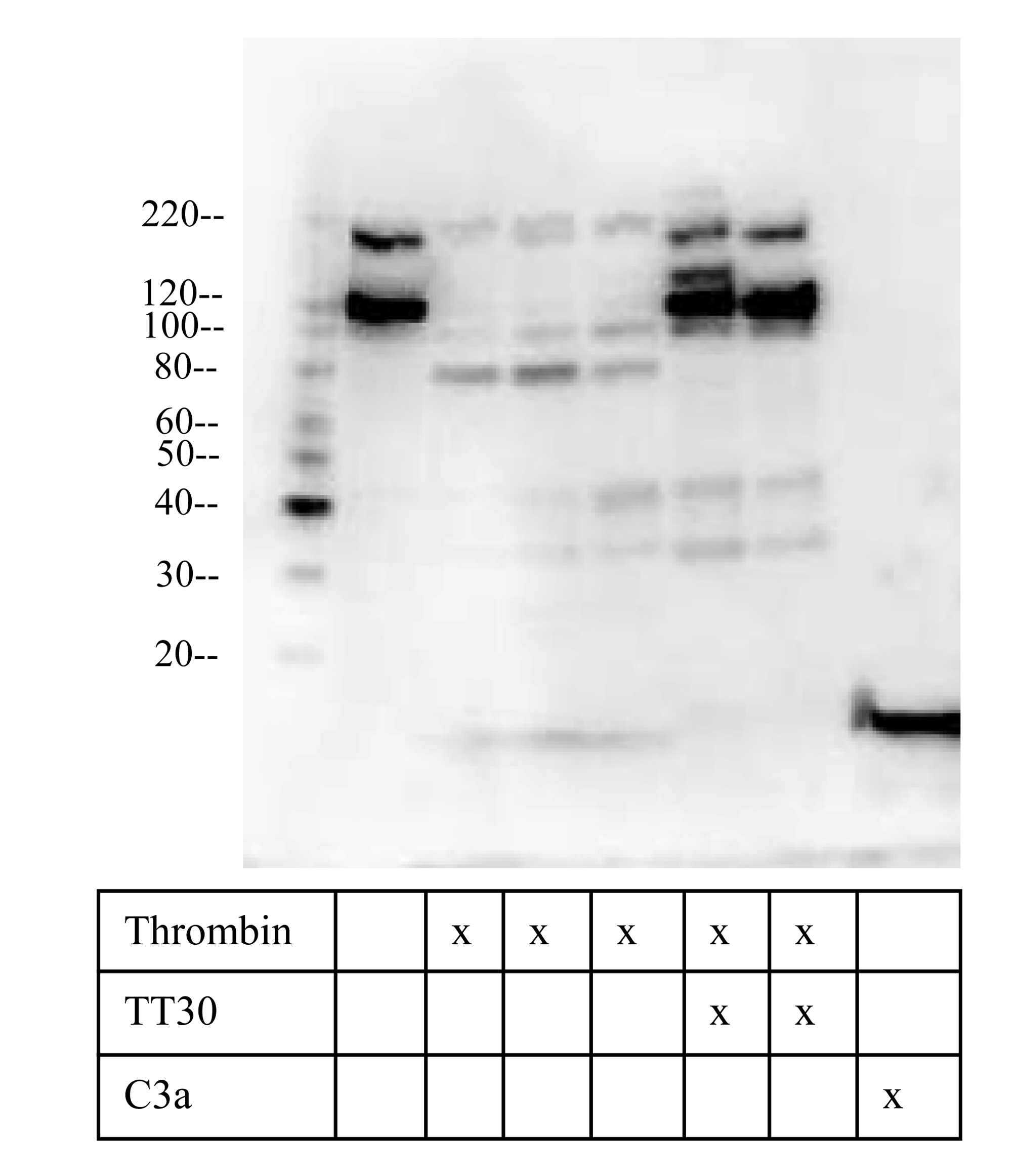

Supplement: Supplementary Figure 2 — Immunogenicity and molecular weight of the C3α fragment generated by thrombin mediated C3 cleavage. Supernatants of cells treated with thrombin, or TT30 were run in parallel with purified mouse C3a (Comptech). The low molecular weight bands produced in the presence of thrombin run at the same molecular weight as purified C3a. [file Image_2.tif]
